# Angiotensin II: A Multimodal Approach to Vasoplegia in a Cardiac Setting

**DOI:** 10.14797/mdcvj.576

**Published:** 2021-11-03

**Authors:** William Sovic, Christo Mathew, Britton Blough, Kara-ann Monday, Teena Sam, Hira Zafar, Cesar Guerrero-miranda, Melody Sherwood, Christopher Hebert, Shelley Hall, Amit Alam

**Affiliations:** 1 Baylor University Medical Center, Dallas, Texas, US; 2 Texas A&M University College of Medicine, Bryan, Texas, US

**Keywords:** Angiotensin II, shock, heart failure, vasoplegia

## Abstract

Patients experiencing vasoplegia, a type of distributive shock, have limited options when conventional vasopressors are not appropriate or sufficient. This is especially true for patients with cardiac dysfunction, whether after heart transplant or ventricular assist device (VAD) implantation. Angiotensin II has been used in various clinical settings for distributive shock; however, its role in patients after orthotopic heart transplant or VAD implantation is not well studied. We present two cases where angiotensin II played a vital role in correcting vasoplegia for critical cardiac patients.

## Introduction

Vasoplegia, especially after cardiopulmonary bypass, is a challenging scenario commonly encountered by the heart failure community. Refractory vasoplegia is associated with high mortality.^1^ The pathophysiology of vasoplegia is multifactorial and involves hyporesponsiveness to vasopressors. This is due to multiple intrinsic vasodilatory pathways including increased nitric oxide production, desensitization of G-protein-coupled receptors (such as adrenergic, vasopressin 1, and angiotensin type 1 receptors) in vascular smooth muscle, and corticosteroid insufficiency and resistance in patients with critical illnesses.^2^ Acute management includes pressors and treatment of the suspected underlying etiology. Angiotensin II (ANG2) is currently approved by the US Food and Drug Administration (FDA) for use in distributive shock. The role of ANG2 in patients with profound nonseptic shock, especially in patients with advanced heart failure, is not well studied.

The Angiotensin II for the Treatment of High-Output Shock (ATHOS 3) trial established the use of ANG2 for vasodilatory shock and showed that ANG2 effectively increased blood pressure in patients with vasodilatory shock who did not respond to high doses of conventional vasopressors.^1^ Furthermore, a post-hoc analysis of this trial found that patients with vasoplegic shock after cardiac surgery with cardiopulmonary bypass (CPB) rapidly responded to ANG2, with improvement in blood pressure and decreasing catecholamine requirements in as little as 3 hours after initiation.^3^ Importantly, none of the patients in this analysis who received ANG2 had venous or arterial thrombotic events, the most feared adverse event of this drug. However, despite this optimistic analysis, the study lacked statistical power due to the low number of patients and because the original trial was not powered to detect mortality differences.

The use of ANG2 for patients with post-CPB vasoplegia offers a theoretical physiologic benefit. The CPB procedure allows blood to bypass the pulmonary circulation and therefore the angiotensin-converting enzyme (ACE) bound to lung endothelium, which converts ANG1 to ANG2. It is not known if additional unique pathophysiologic mechanisms occur in patients with vasoplegia after heart transplant or if placement of a left ventricular assist device (LVAD) may especially warrant ANG2.

We describe two encounters of successful use of ANG2 in patients with advanced heart failure.

## Case 1

A 59-year-old Caucasian male, United Network for Organ Sharing (UNOS) status 4 secondary to hypertrophic cardiomyopathy, underwent orthotopic heart transplantation (OHT) at our institution (***Table 1***). Immediately after the procedure, the patient developed hypotension (56 mm Hg mean arterial pressure) refractory to norepinephrine (0.15 mcg/kg/min), vasopressin (0.04 units/min), and epinephrine (0.1 mcg/kg/min). His blood pressure did not improve despite the use of methylene blue and the initiation of veno-arterial extracorporeal membrane oxygenation (ECMO). Vasoplegia from severe primary graft dysfunction was the determined etiology of the patient’s hypotension given a reduced systemic vascular resistance of 542 (dyne*sec)/cm^5^ with severely reduced left ventricular function based on intraoperative echocardiogram (echo). The patient had no known risk factors for graft dysfunction and no history of autoimmune disease nor detectable pretransplant human leukocyte antigen antibody titers. There was no concern for inadequate immunosuppression or rapid metabolizing of immunosuppression because the patient’s ejection fraction normalized on echo by postoperative day 7.

**Table 1:** Patients with advanced heart failure started on angiotensin II (ANG2). MAP: mean arterial pressure


	PATIENT ONE	PATIENT TWO

**Age (years)**	59	44

**Gender**	Male	Male

**Race**	Caucasian	African American

**Primary acute illness**	Primary graft dysfunction	Right heart failure post left ventricular assist device

**MAP initiation for ANG2 (mm Hg)**	56	45

**Duration of ANG2 (hours)**	24	60

**Peak dose of ANG2 (ng/kg/min)**	20	30

**Patient outcome**	Discharged to rehab facility	Discharged home, subsequent orthotopic heart transplantation


ANG2 was initiated, and over the course of 24 hours after starting the medication, the patient’s hemodynamics greatly improved, and he was able to be weaned off inotropes and pressors with eventual discontinuation of ECMO support (***Table 2***). In fact, by 5 hours after ANG2 initiation, the patient had been weaned off norepinephrine and was receiving epinephrine at 0.03 mcg/kg/min and vasopressin at 0.03 units/min. The patient was ultimately discharged to a rehab facility. The ejection fraction of his allograft normalized before discharge, and post-transplant surveillance endomyocardial biopsy showed no evidence of rejection.

**Table 2:** Hemodynamic parameters pre- and post-initiation of angiotensin II (ANG2). MAP: mean arterial pressure; RA: right atrium; PA: pulmonary artery; PCWP: pulmonary capillary wedge pressure; SVR: systemic vascular resistance; CI: cardiac index; NED: norepinephrine equivalent dose


	MAP	RA	PA	PCWP	SVR	CI	NED

**Immediately pre-initiation of ANG2**							

Patient one	56 mm Hg	14 mm Hg	24/17 mm Hg	14 mm Hg	542 (dyne*sec)/cm^5^	3.10	0.35

Patient two	45 mm Hg	20 mm Hg	61/31 mm Hg	27 mm Hg	319 (dyne*sec)/cm^5^	2.88	0.3

**~12 hours post-initiation of ANG2**							

Patient one	85 mm Hg	8 mm Hg	40/18 mm Hg	15 mm Hg	1185 (dyne*sec)/cm^5^	2.6	0.105

Patient two	65 mm Hg	6 mm Hg	54/17 mm Hg	16 mm Hg	875 (dyne*sec)/cm^5^	2.5	0.1


## Case 2

A 44-year-old African American male with LVAD (HeartMate 3) implantation 5 months prior presented with profound right ventricular failure refractory to speed adjustments and inotrope optimization (***Table 1***). Upon examination, the patient had volume overload and required aggressive fluid removal hampered by persistent hypotension. Right heart catheterization revealed significantly decreased systemic vascular resistance of 319(dyne*sec)/cm^5^. Thus, ANG2 was initiated despite concerns of the potential thromboembolic risk posed by the patient’s LVAD.

The patient’s vasoplegia started to improve within 2 hours of initiating ANG2. Because of hemodynamic progress (***Table 2***), he was able to complete fluid removal with continuous renal replacement therapy, and his symptoms resolved. One month after discharge, the patient underwent OHT.

## Discussion

Our cases demonstrate the successful use of ANG2 in two patients with advanced heart failure, a condition that was excluded from the ATHOS-3 trial. Theoretically, ANG2 may be of particular benefit after CPB because the procedure bypasses pulmonary circulation and therefore bypasses ACE, which is required for the conversion of ANG1 to ANG2.^4^ Furthermore, like the post-cardiac-surgery patients in the post-hoc analysis of ATHOS-3, our patients did not experience any arterial or venous thrombotic events, although both required anticoagulation for their respective mechanical assist devices. Both were successfully discharged from the hospital with uneventful follow-up 6 months to date.

We are living in an exciting time in cardiovascular medicine and especially in addressing heart failure. Current guideline-based evidence supports multiple medications to target different pathways in patients with heart failure with reduced ejection fraction (HFrEF): ACE inhibitors, beta blockers, angiotensin receptor-neprilysin inhibitors, mineralocorticoid receptor antagonists, and more recently the sodium-glucose co transporter-2 inhibitors. Ongoing research shows that simultaneously targeting different mechanisms has morbidity and mortality benefits.^5^ Similarly, three endogenous hormones are heightened in a shock state: catecholamines, vasopressin, and angiotensin II. Like the drugs that treat HFrEF through different mechanisms, ANG2 represents a single treatment in a multimodal therapeutic approach for vasoplegia.^6^

Our cases specifically show a potential benefit of ANG2 for vasoplegia following OHT and LVAD procedures. The success of ANG2 in patients, like ours, receiving advanced heart failure therapies should be authenticated through future research and randomized controlled trials using ANG2 to optimize outcomes for advanced heart failure patients in the intensive care unit who are admitted for severe vasoplegia.^7^
